# An Anti-Suicide Club

**Published:** 1909-01-16

**Authors:** 


### The Hospital

A Journal of

The Medical Sciences and Hospital Administration.

New Series. No. 98, Vol. IV. [No. 1170, Vol. XLV.] SATURDAY, JANUARY 16, 1909.

### An Anti-Suicide Club.

The Suicide Club, as every reader of Robert Louis Stevenson knows quite well, came to a sudden end many years ago, through the agency of Prince Florizel of Bohemia and his resourceful aide-de-camp, Colonel Geraldine. It will be remembered that this organisation provided a graceful and gentlemanly means of exit for those members of the leisured classes who, from *tædium vitæ*, financial disaster or other sufficient motive, sought to destroy themselves, but lacked the opportunity or the courage requisite for doing so without scandal and without misadventure. Having paid a somewhat heavy entrance-fee, the members met nightly at the club-rooms, and, under the inventive stage-management of the President, decided by the turn of the cards who among them should have the privilege at the close of the evening of ushering a fellow-member through the portals of death. We are reminded of this ingenious and exciting tale by an account lately issued of the first year's working of the "Anti-Suicide Bureau," which was founded in 1907 by the Salvation Army. The need and use of this novel institution seems to have been proved already, and it promises to become a permanent light-house amidst the dark and troubled eddies of London life.

Suicide is on the increase, and there can be little doubt that a large number of those unfortunates who take their own lives are, for all practical purposes, in the possession of their senses, both when they contemplate suicide and at the time of the act itself. The verdict of "suicide during temporary insanity" is as often as not a conventional *façon de parler*, pronounced by a kind-hearted jury with the idea of sparing the feelings of relatives, securing burial in consecrated ground, and frustrating that legitimate confiscation of insurance-money which some Life Offices visit upon a verdict of *felo de se*. This point was raised quite lately by a juryman at an inquest in Dr. Waldo's court, and an interesting exchange of views took place between the City Coroner and several members of his jury. Dr. Waldo holds the opinion that in those cases of suicide wherein no reasonable evidences of insanity, but rather the contrary, are forthcoming, it is the jury's duty to suppress sentiment, and at whatever cost to bring in the harsher verdict. His justification for this advice is the alarming increase of late years in the number of suicides, which he not unreasonably considers is encouraged by the prevalent custom of indiscriminate verdicts of "temporary insanity." The knowledge that *felo de se*, with its attendant consequences to those dear to him, will inevitably follow his act, might well give pause to a sane man hovering upon the brink of self-destruction. With such a man, crushed by misfortunes or unnerved by fear or worry, the contemplation of suicide as the best way out of it all may merge rapidly into a strong desire, and that again into a fixed intention; but, unlike the insane man, he is seldom spurred on to the fatal act by an overmastering irresistible impulse. Quite small considerations, more trifling than the prospect of a suicide's grave, less weighty than the forfeiture of insurance money, may put him off. Therefore, if the verdict of *felo de se* be a deterrent to self-murder, however occasionally or however remotely, it is right to make full use of it whenever sanity cannot be disproved; and this should not be carried out capriciously or locally or by fits and starts, but as the settled uniform practice of Coroner's Courts throughout the country.

It is estimated that more than 3,000 persons commit suicide each year in these islands. How many more contemplate suicide is a matter for conjecture: but their number must be very large. As for those who attempt to end their lives, and fail to do so, because of ignorance or half-heartedness, or through the intervention of providence, as often as not in the form of doctor or policeman—their number also is lamentably large. It is of course primarily for the second class—those who are contemplating suicide—that the Bureau exists. It seeks to reach these persons, and by sympathy, expert advice or material assistance to dissuade them from their purpose. Last year, we learn, over 1,000 men sought the help of the Bureau, and it is stated that the large majority of the applicants are now restored to their normal condition. More than once during the past year we considered the subject of suicide in these columns, mainly in its scientific aspects. The records of this new department of the Salvation Army provide additional data of a somewhat rough and ready character for the ætiological study of suicide. Thus we learn that financial embarrassment or hopeless poverty was the cause in about half the cases; accidents, sickness, and kindred misfortunes in 21 per cent. Drink, drugs, and disease account for 11 per cent.; melancholia proceeding from loneliness and other causes for 9 per cent.; forgery, embezzlement, and other crimes for 5 per cent. The Bureau deals mainly with the middle-classes, but many professional men have also sought help. Thus, among the applicants for advice or assistance have been clergymen, military officers, doctors, actors, solicitors, journalists, architects, surveyors, and artists. The Bureau has done good work. Its aim is truly philanthropical; and its methods are practical, sensible, and, in the broadest meaning of the word, scientific.

